# Editorial: Improving clinical practice for the diagnosis and management of patients with leptomeningeal metastasis

**DOI:** 10.3389/fneur.2024.1367547

**Published:** 2024-03-08

**Authors:** Giuseppe La Rocca, Edoardo Mazzucchi, Roberto Altieri, Vittorio Orlando, Gianluca Galieri

**Affiliations:** ^1^Institute of Neurosurgery, Fondazione Policlinico Universitario A. Gemelli IRCCS, Catholic University, Rome, Italy; ^2^Department of Neurosurgery, Mater Olbia Hospital, Olbia, Italy; ^3^Department of Neurosurgery, “San Carlo” Hospital, Potenza, Italy

**Keywords:** leptomeningeal metastasis, CSF parameters, prediction models, GTR, molecular target therapy

Leptomeningeal metastasis (LM) represents the spread of malignant cells into the subarachnoid space, pia, and arachnoid mater through hematogenous spread, perivascular or perineural dissemination along peripheral nerves, or direct expansion of parenchymal cerebral metastases into the cerebrospinal fluid (CSF). Our Editorial on the Special Research Topic entitled “*Improving Clinical Practice for the Diagnosis and Management of Patients with Leptomeningeal Metastasis*” provides an overview about this neuro-oncological topic from a multidisciplinary point of view. Four articles are included in this Research Topic.

In the scientific literature, the reported incidence of leptomeningeal metastases ranges from ~1–15%. However, this figure may significantly increase in postmortem studies, a discrepancy attributed to advancements in diagnostic imaging techniques improving detection, coupled with enhanced patient survival resulting from more efficacious therapeutic interventions.

The prognosis for patients with leptomeningeal metastases is grim, with survival ranging from a mere 6–8 weeks without intervention to an extension of survival to 2–6 months with targeted tumor-specific treatments, including intrathecal chemotherapy, systemic chemotherapy, and radiotherapy. Optimal outcomes can be achieved with early detection and the strategic application of individualized therapeutic strategies.

LM symptoms depend on brain, cranial nerve, and spinal cord involvement and typically include headache, nausea and vomiting, diplopia, facial weakness, changes in hearing, gait difficulties, paresthesia, neck and back pain, mental changes and cranial neuropathies. However, these symptoms are nonspecific and may be present in other conditions.

Articles in this study explore valuable models for enhancing LM diagnosis and analyze potential treatments with an increasing focus on immunohistochemistry and molecular tumor characteristics, paving the way for more personalized therapeutic approaches. Diagnosing LM poses challenges relying on clinical manifestations, CSF cytopathology, and neuroimaging findings. The EANO-ESMO guidelines establish four evidence levels for LM diagnosis: confirmed, probable, possible, and lack of evidence.

A retrospective analysis by Bönig et al. highlights the significance of CSF examination in cases where MRI is negative and CSF cell count is normal. Findings show that patients with hematological malignancies exhibit higher CSF cell counts, CSF lactate concentrations, CSF total protein levels, and CSF-serum albumin quotient (QAlb) values, even if signs of LM are not evident in MRI findings.

Key CSF findings for hematological malignancies include elevated CSF cell count in 92% of cases, increased CSF lactate levels in 48%, elevated CSF glucose levels in 54%, escalated CSF total protein in 89%, and blood-CSF barrier dysfunction in 92%. Patients with solid malignancies showed predominant CSF findings, including increased CSF cell count in 79%, heightened CSF lactate levels in 68%, elevated CSF glucose levels in 57%, escalated CSF total protein in 82%, and blood-CSF barrier dysfunction in 80%.

In 2022, Gao T. et al. developed two diagnostic prediction models for solid tumor patients to improve LM diagnosis and avoid unnecessary examinations or lumbar puncture (LP). Models A and B include predictors such as skull metastasis, active brain metastasis, progression of extracranial disease, the number of extracranial organs involved, and symptoms. Model B incorporates two additional predictors, protein and glucose levels in CSF. The AUC values for models A and B were 0.812 and 0.901, respectively, indicating improved diagnostic accuracy compared to initial MRI and LP. The models exhibit a net clinical benefit in medical decisions and in avoiding unnecessary interventions for patients with LM.

Gao Y. et al. reported a rare case of intracranial solitary fibrous tumor (iSFT), first categorized as hemangiopericytoma cell tumor (HCP) in the previous brain tumor classifications. He was treated with a combined multiple approach over approximately 14 years, emphasizing the importance of surgical excision supplemented by stereotactic radiotherapy, gamma knife, stereotactic radiosurgery, molecular targeting, and immunotherapy. As survival times for SFT extend, meticulous long-term follow-up becomes imperative.

Another study by de Bernardi et al. reported a sustained response exceeding 2 years in a patient with HER2-positive metastatic breast cancer with LM. The extended response was achieved through sequential systemic and intrathecal anti-HER2 therapies, highlighting the potential for durable survival with appropriate treatment of HER2-positive LM.

In conclusion, LM diagnosis relies on clinical evaluation, MRI findings, and CSF examination. Prediction models and specific CSF parameters can improve diagnosis and allow for early treatment.

The inclusion of liquid biopsy (derived from either blood or CSF) in the diagnostic process is essential for early detection of LM. Wijaya et al. (1) systematically examined the diagnostic efficacy of circulating tumor DNA (ctDNA) obtained from CSF in comparison to plasma ctDNA in LM patients. Their findings affirm that liquid biopsies possess superior diagnostic efficacy for detecting genomic alterations when utilizing CSF-derived samples compared to plasma. Moreover, CSF-derived ctDNA provides a more comprehensive depiction of the molecular landscape associated with LM. Surgical excision with GTR is the first-line treatment, but a multimodal approach with radiosurgery, radiotherapy, chemotherapy, and specific molecular targeted therapy is necessary for increased survival and reduced disease progression. The future of therapeutic approaches is evolving toward personalized strategies tailored to individual patients, necessitating the identification of increasingly specific molecular targets.

## Author contributions

GL: Conceptualization, Writing – original draft, Writing – review & editing. EM: Conceptualization, Investigation, Writing – review & editing. RA: Data curation, Methodology, Supervision, Writing – original draft. VO: Data curation, Investigation, Methodology, Writing – original draft. GG: Conceptualization, Writing – original draft, Writing – review & editing.
